# Surgical Treatment of Spontaneous Intracranial Hypotension: Clinical Characteristics and Outcomes in a Surgically Treated Cohort of Type 1 and Type 3 Leaks

**DOI:** 10.3390/jcm15134972

**Published:** 2026-06-26

**Authors:** Woo-Seok Ha, Hyun Woong Mun, Soomi Cho, Chang Kyu Lee, Dong Ah Shin, Seong Yi, Keung Nyun Kim, Min Kyung Chu, Yoon Ha

**Affiliations:** 1 Department of Neurology, Severance Hospital, Yonsei University College of Medicine, 50-1 Yonsei-ro, Seodaemun-gu, Seoul 03722, Republic of Koreasoomicho@yuhs.ac (S.C.); 2 Department of Neurosurgery, Spine and Spinal Cord Institute, Severance Hospital, Yonsei University College of Medicine, 50-1 Yonsei-ro, Seodaemun-gu, Seoul 03722, Republic of Korea; askl00@yuhs.ac (H.W.M.); nscklee@yuhs.ac (C.K.L.); cistern@yuhs.ac (D.A.S.); yiseong@yuhs.ac (S.Y.); knkim@yuhs.ac (K.N.K.)

**Keywords:** intracranial hypotension, cerebrospinal fluid leak, CSF-venous fistula, headache, subdural hematoma

## Abstract

**Background/Objectives**: Spontaneous intracranial hypotension (SIH) is a functionally limiting condition caused by cerebrospinal fluid leakage. This study aims to evaluate the clinical outcomes of surgical management based on precise leak localization and to describe the characteristics of a surgically treated SIH cohort. **Methods**: We enrolled 23 patients who underwent surgical treatment for SIH between March 2024 and November 2025. Clinical outcomes included maximum headache severity and total daily upright hours. Radiologic outcomes were evaluated using the Bern score and the resolution of spinal extradural fluid at 2 months postoperatively. **Results**: The cohort comprised 19 patients (82.6%) with Type 1 and four patients (17.4%) with Type 3 leak. Exploratory subgroup analyses suggested that patients with Type 3 leak were significantly older (mean 65.3 vs. 38.2 years, *p* < 0.01) with lower thoracic leak (*p* = 0.02) compared to Type 1 patients. In the 20 patients who completed follow-up, significant improvements were observed in maximum headache intensity (Numeric Rating Scale 4.6 to 1.4, *p* < 0.01), daily upright time (3 to 12 h, *p* < 0.01), and Bern score (3.4 to 0.9, *p* < 0.01). Postoperative rebound headache occurred in 52.2% of patients. Complete resolution of spinal epidural fluid was achieved in 87.5% of Type 1 patients and normalization of the Bern score was achieved in all Type 3 patients. **Conclusions**: Surgical intervention based on precise leak localization offers substantial clinical and radiological benefits for SIH patients refractory to conservative management. These findings support a treatment-oriented approach based on precise leak localization in patients with SIH.

## 1. Introduction

Spontaneous intracranial hypotension (SIH) is a clinically significant disorder caused by cerebrospinal fluid (CSF) leakage from the spinal canal [1]. Its clinical manifestations are thought to arise from downward traction on pain-sensitive dural structures due to posture-dependent CSF outflow, particularly in the upright position. Although most patients initially present with acute orthostatic headache, diagnostic complexity increases over time, as more than one-third develop non-orthostatic or even non-headache symptoms [2]. While often considered benign, SIH can lead to substantial morbidity, long-term neurological impairment such as superficial siderosis or dementia [3,4], and, in rare cases, altered consciousness or coma [5], emphasizing the need for accurate diagnosis and timely intervention.

The advent of high–temporal resolution spinal modalities, such as digital subtraction myelography (DSM) or dynamic CT myelography (CTM), has changed the management of SIH [6,7]. These invasive techniques allow for precise localization of the leakage site, facilitating a paradigm shift from empiric, non-targeted epidural blood patches (EBPs) to definitive, site-specific interventions [8]. In addition, advances in spinal imaging have enabled the classification of CSF leaks according to their underlying pathophysiology, including Type 1 (ventral dural tear), Type 2 (lateral leak associated with meningeal diverticula), Type 3 (CSF-venous fistula), and Type 4 (indeterminate) [1]. Because diagnostic approaches and treatment strategies differ substantially among these leak subtypes, accurate classification has become increasingly important in contemporary SIH management. While targeted EBPs remain a primary option, surgical repair has emerged as a curative treatment for patients with refractory symptoms or specific pathologies that are amenable to direct closure.

Despite these changes, the adoption of surgical management for SIH remains limited in Asian regions, and literature describing surgical outcomes in this population is scarce [9]. Our institution has implemented a structured diagnostic and therapeutic protocol to identify optimal candidates for surgical intervention. This study aims to evaluate the clinical outcomes of surgical management based on precise leak localization and to describe the clinical characteristics of a surgically treated cohort of patients with Type 1 and Type 3 CSF leaks.

## 2. Materials and Methods

### 2.1. Study Participants

This retrospective study was conducted at a single tertiary spine center, covering the period from March 2024 to November 2025. The study protocol was approved by the institution’s Institutional Review Board (IRB No. 4-2024-1323), and the requirement for informed consent was waived due to the retrospective nature of the study using medical records. The inclusion criteria comprised individuals treated surgically for SIH in our center. Patients diagnosed with SIH at our institution but who underwent surgical treatment at other facilities were excluded. The diagnosis of SIH was established according to the International Classification of Headache Disorders, 3rd edition criteria [10]. Demographic data, including age, sex, body mass index, date of symptom onset, and history of attempted EBP procedures prior to referral or surgery, were collected for all patients. Upon initial presentation, all patients completed questionnaires, either via mobile devices or on paper, recording the maximum intensity of orthostatic headache using the Numerical Rating Scale (NRS) and the total daily upright hours (calculated as the average over the preceding three days), following a recent consensus guideline [11].

### 2.2. Preoperative Evaluation and Surgical Indications

**Figure 1:**
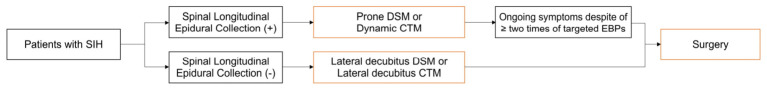
Diagnostic and treatment algorithm for spontaneous intracranial hypotension. SIH, spontaneous intracranial hypotension; DSM, digital subtraction myelography; CTM, CT myelography; EBP, epidural blood patch.

The patient selection process for surgery followed the institutional standard of care diagnostic and treatment algorithm (Figure 1) [12]. All patients with clinically suspected SIH underwent initial assessments, including brain MRI with contrast enhancement, spinal MR myelography, and whole spine CT with three-dimensional reconstruction. Patients were classified according to the dedicated classification system of spontaneous spinal CSF leaks [1]. Type 1 leak (ventral dural tear) was confirmed by DSM or dynamic CTM in the prone position. Type 3 leak (CSF-venous fistula) was confirmed by DSM or CTM in the lateral decubitus position. No patients with Type 2 (lateral leak associated with meningeal diverticula) or Type 4 (indeterminate) underwent surgical treatment during the study period at our institution, and thus only Type 1 and Type 3 cases were included [1].

Surgical indications differed by subtype. For Type 1 leak, surgery was recommended if the leak site was identified and symptoms persisted despite at least two trials of targeted EBPs. For Type 3 leak, surgical treatment was recommended immediately upon diagnosis without requiring prior EBP trials, given the limited efficacy of EBP in this subtype based on its underlying pathophysiology. All brain MRI scans were evaluated using the Bern score, a validated MRI-based scoring system for quantifying the radiologic severity of SIH [13]. In cases where SLEC was observed on MR myelography, the presence of an “organized SLEC”, defined as a collection with sharply demarcated, convex edges confined to the ventral aspect, was also recorded [14]. CSF opening pressure was measured in the lateral decubitus position for all patients when undergoing dynamic myelography.

### 2.3. Surgical Techniques

For Type 1 leaks, laminotomy or partial hemilaminectomy was performed to access the ventral aspect of the thecal sac, followed by direct ventral dural repair. Dorsal durotomy was then carried out, followed by sectioning of the dentate ligament at the index level to allow gentle mobilization of the spinal cord and facilitate access to the ventral dural defect. Repair techniques included the use of autologous muscle grafts as well as adjunctive agents, such as TachoComb^®^ (Takeda Pharmaceutical Company, Tokyo, Japan) or Hemopatch^®^ (Baxter Healthcare SA, Zurich, Switzerland). For Type 3 leaks, a partial hemilaminectomy was performed to expose the dura and surrounding venous structures. Once the CSF-draining venous channel was identified, indocyanine green (ICG) fluorescence angiography was conducted intraoperatively to confirm venous flow. The fistula was then ligated directly using bipolar coagulation and microsurgical clipping. A follow-up ICG angiography was performed to confirm the absence of residual flow.

### 2.4. Intraoperative and Postoperative Evaluation

Intraoperative data, including operative time and estimated blood loss, were collected. For Type 1 leak, the maximum diameter of the dural tear was recorded in millimeters. Postoperative outcomes, including neurological deterioration and other complications, were documented. All patients were evaluated by a headache specialist for the presence of rebound headache suggestive of intracranial hypertension. Rebound headache was diagnosed clinically based on the development of a new postoperative headache pattern that differed from the preoperative SIH headache, typically occurring immediately after surgery or within the first postoperative day. Clinical features included holocephalic pain and worsening in the recumbent position or during Valsalva maneuvers. The diagnosis was based on clinical assessment, and routine confirmation with CSF opening pressure measurement was not performed.

Follow-up evaluations were conducted at 2 months postoperatively. These included a brain MRI to reassess the Bern score and questionnaires regarding headache intensity and daily upright hours. Additionally, patients with Type 1 leak underwent follow-up MR myelography to evaluate the resolution of SLEC. Radiological resolution was defined according to the specific leak subtype: for Type 1, it was defined as the complete disappearance of SLEC on follow-up MR myelography, whereas for Type 3, it was defined as the normalization of the Bern score on brain MRI.

### 2.5. Statistical Analysis

Analyses of demographic characteristics, intraoperative findings, and complications included the entire surgically treated cohort. However, the analysis of pre- and postoperative clinical and radiologic outcomes was restricted to patients who completed the 2-month follow-up evaluation. Continuous variables were presented as means with standard deviations (SD) if normally distributed (verified by the Kolmogorov–Smirnov test) or as medians with interquartile ranges (IQR) if non-normally distributed. Categorical variables were expressed as frequencies and percentages. Comparisons between Type 1 and Type 3 groups were performed using the Mann–Whitney U test, Chi-square test, or Fisher’s exact test, as appropriate. Given the small sample size, the Wilcoxon Signed-Rank Test was used to assess changes in pre- and postoperative measures in the entire cohort. Statistical significance was defined as a two-sided *p*-value < 0.05. All analyses were performed using SPSS Statistics for Windows, version 28.0 (IBM Corp., Armonk, NY, USA).

## 3. Results

### 3.1. Patient Demographics

A total of 23 patients were included in the study. The mean age at presentation was 42.43 ± 14.54 years, with 9 males and 14 females. The cohort consisted of 19 patients (82.6%) with Type 1 leak and 4 patients (17.4%) with Type 3 leak (Table 1). Patients with Type 3 leak were significantly older than those with Type 1 leak (mean age 65.3 vs. 38.2 years, *p* < 0.01). While the majority of Type 1 leaks were located in the upper thoracic spine (78.9%), Type 3 fistulas were predominantly found in the lower thoracic spine (75.0%) (*p* = 0.02). Given the limited number of patients with Type 3 leaks, these observations should be interpreted cautiously. The mean CSF opening pressure was 128.7 mmH_2_O, with no significant difference observed between the groups (*p* = 0.37). CSF opening pressure data were not recorded in two patients. Notably, among the 21 patients, only one patient presented with a pressure below 60 mmH_2_O. Among the 19 patients with Type 1 leak, 14 (73.7%) exhibited an “organized SLEC” on preoperative MR myelography. Some patients underwent both DSM and dynamic CTM as part of the diagnostic workup; therefore, the numbers reported for each modality are not mutually exclusive.

### 3.2. Intraoperative Findings

The mean operative time was significantly shorter in the Type 3 group compared to the Type 1 group (95.5 vs. 151.7 min, *p* < 0.001). Intraoperative blood loss did not differ significantly between the groups. In Type 1 patients, the median size of the dural defect was 3 mm (IQR 2–3).

### 3.3. Postoperative Outcomes

**Figure 2:**
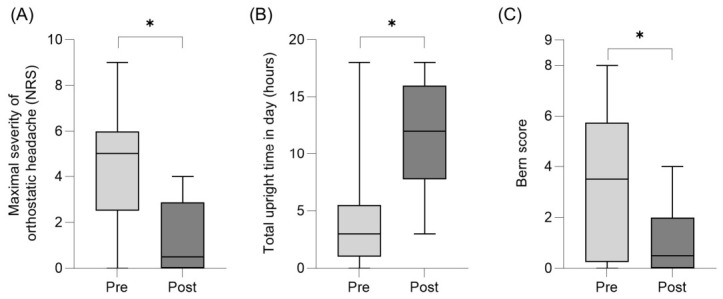
Box-and-whisker plots for comparing pre and postoperative clinical and radiologic outcomes. (**A**) Maximal headache severity assessed by the Numeric Rating Scale (NRS). (**B**) Total daily upright time in hours, and (**C**) Bern score. * *p* < 0.01.

Of the 23 patients, 20 completed the 2-month follow-up evaluation (Table 2 and Figure 2). Three patients with Type 1 leak were excluded from the outcome analysis: two had not yet reached the 2-month postoperative time point, and one was unable to undergo follow-up imaging and assessment due to persistent non-cooperation related to frontotemporal brain sagging syndrome.

In the follow-up cohort (n = 20), significant improvements were observed across all clinical parameters. The mean maximum headache intensity (NRS) decreased from 4.6 (±2.7) preoperatively to 1.4 (±1.6) postoperatively (*p* < 0.01). The median daily upright time increased significantly from 3 h (IQR 1–4.5) to 12 h (IQR 5–16) (*p* < 0.01). The mean Bern score also improved significantly from 3.4 (±2.8) to 0.9 (±1.1) (*p* < 0.01), indicating radiologic resolution of intracranial hypotension.

Among the 16 patients with Type 1 leak, complete resolution of SLEC was achieved in 14 patients (87.5%). In the two cases where SLEC persisted, one involved a failure to identify the dural tear intraoperatively, while the other involved a persistent collection despite repair. Both instances occurred during the early phase of our learning curve. Retrospectively, we attribute these outcomes to the ventral dural defects being obscured by pseudodura, arachnoid scarring or adhesions, which prevent adequate dissection and complete visualization of the defects. Revision surgery is currently being considered for both patients.

### 3.4. Complications

Postoperative complications are detailed in Table 1. Rebound headache suggestive of intracranial hypertension occurred in 12 patients (52.2%), all of whom were in the Type 1 group. The headaches were predominantly holocephalic and typically worsened when lying down or during Valsalva maneuvers such as coughing. All cases were successfully managed with oral acetazolamide, which was tapered off within one month in most patients. Transient postoperative neurological deterioration was observed in three patients (13.0%), all of whom experienced temporary lower extremity weakness. Notably, all affected individuals recovered completely within six months postoperatively. Surgical revision was required in one case (4.3%) due to a postoperative spinal epidural hematoma necessitating evacuation.

### 3.5. Illustrative Case 1–Type 1 Leak

**Figure 3:**
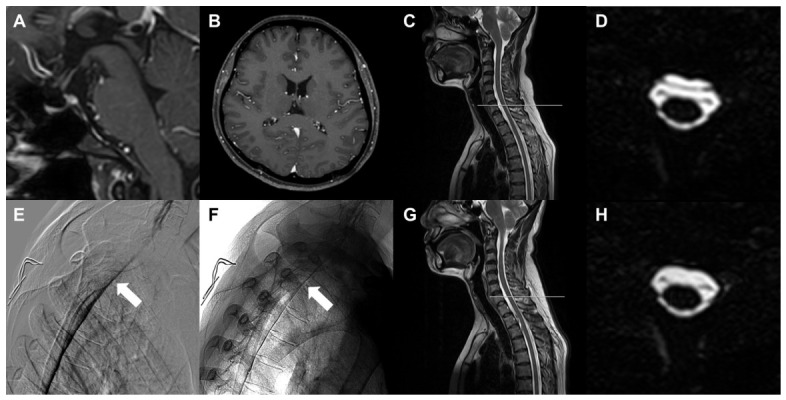
A case of a 55-year-old female with Type 1 leak. (**A**,**B**) Preoperative brain MRI demonstrating normal intracranial appearances with a Bern score of 0, despite the patient’s persistent symptoms. (**C**,**D**) Preoperative MR myelography showing an organized ventral spinal longitudinal extradural collection (SLEC) extending from C5 to T4. (**E**,**F**) Digital subtraction myelography images localizing the rapid ventral CSF leakage at the T2–3 level (white arrows). (**G**,**H**) After two months of surgery, an MR myelography confirming complete resolution of the SLEC.

A 55-year-old woman presented with a 7-year history of non-orthostatic headache, and neurologic examination was unremarkable. She had previously received numerous EBPs between 2017 and 2024, each providing only transient relief. Although her initial brain MRI at symptom onset in 2017 indicated a Bern score of 5, preoperative imaging after referral revealed normalization of the Bern score to 0 (Figure 3A,B). However, spinal MR myelography demonstrated a persistent, organized SLEC extending from C5 to T4 (Figure 3C,D). The CSF leak was localized to the ventral T2–3 level using DSM and dynamic CTM (Figure 3E,F). The patient subsequently underwent a targeted laminotomy and direct dural repair using an autologous muscle graft reinforced with Hemopatch^®^ (Supplementary Video Clip S1). Postoperatively, she experienced complete resolution of headache symptoms. At the two-month follow-up, spinal MR myelography confirmed complete resolution of SLEC (Figure 3G,H).

### 3.6. Illustrative Case 2–Type 3 Leak

**Figure 4:**
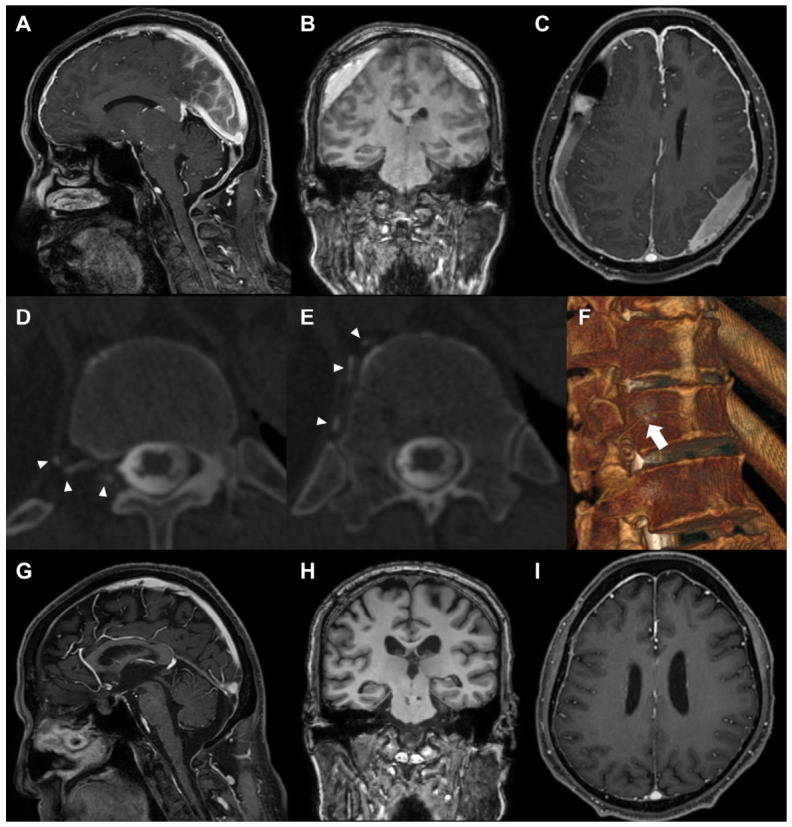
A case of 67-year-old male with Type 3 leak. (**A**–**C**) Preoperative brain MRI demonstrating a bilateral chronic subdural hematoma, pachymeningeal enhancement, and venous engorgement, with a Bern score of 9. (**D**,**E**) Lateral decubitus CT myelography revealing abnormal contrast egress from the right T12–L1 diverticulum into the paraspinal vein, consistent with a CSF–venous fistula. The white arrowheads indicate the draining paraspinal vein opacified through the CSF–venous fistula. (**F**) Three-dimensional reconstructed image showing the fistula (white arrow). (**G**–**I**) Postoperative brain MRI demonstrating a Bern score of 2 and complete resolution of the subdural hematoma.

A 67-year-old man presented with a 4-month history of progressive gait disturbance, orthostatic headache, and cognitive decline. Prior to referral, he had undergone two burr hole drainages for chronic subdural hematoma (SDH) and five EBPs. However, he experienced progressive neurological deterioration due to recurrent SDH (Figure 4A–C).

Spinal MR myelography showed no SLEC but demonstrated multiple spinal diverticula, most prominently at the right T12–L1 level. Lateral decubitus CTM confirmed a CSF–venous fistula at T12–L1 (Figure 4D–F). Surgical ligation of the fistula was performed (Supplementary Video Clip S2). At the two-month follow-up, brain MRI demonstrated resolution of intracranial hypotension-related changes (Figure 4G–I), and the patient showed resolution of all neurological deficits and regained full independence in activities of daily living.

## 4. Discussion

The major findings of this study are as follows: (1) the implementation of a stratified diagnostic and surgical protocol resulted in significant clinical and radiologic improvements in patients with SIH; (2) exploratory observations regarding demographic and anatomical characteristics of Type 1 and Type 3 leaks were generally consistent with previously reported patterns, although these findings should be interpreted cautiously given the small and highly selected cohort.

EBP has been the mainstay of treatment for SIH for several decades [8]. Although our institution continues to utilize EBP as the primary treatment of choice for ventral leaks, a subset of patients inevitably requires surgical intervention. A recent study has highlighted the prognostic importance of the morphology of SLEC; specifically, the resolution rate of SLEC following traditional EBP is reported to be 54% in patients with unorganized SLEC, whereas it drops to 4% in those with organized SLEC [14]. In our study, 73.7% of surgically treated Type 1 patients presented with organized SLEC, which likely explains their poor response to prior EBPs. Furthermore, the formation of neo-membranes associated with chronic spinal CSF leaks may mechanically obstruct the epidural spread of the blood patch or impede the physiological self-repair pathways of the dura [15]. Regarding Type 3 leak, the structural nature of the CSF-venous fistula renders it inherently resistant to EBP, even if transient symptomatic relief is occasionally observed. Consequently, we advocate for a stratified treatment algorithm that establishes surgical indications based on leak type and response to EBP. Future research should focus on developing screening tools to identify surgical candidates earlier in the disease course, thereby minimizing unnecessary procedures.

Since all procedures in this study were performed via open surgery, operative metrics could be descriptively evaluated according to leak subtypes. The mean operative time for Type 1 repairs was approximately 2.5 h, compared to 1.5 h for Type 3 ligations. Repairing a ventral dural tear typically necessitates extensive bony exposure and retraction of the spinal cord to access the ventral defect. In contrast, the treatment of CSF-venous fistula is a relatively focal procedure, requiring only a hemilaminectomy followed by ligation or clipping of the fistula near the nerve root sleeve. This reduced surgical invasiveness may contribute to the lower complication rate observed in our Type 3 group. With the introduction of transvenous embolization for CSF-venous fistula [16], a recent meta-analysis involving 321 patients showed that both surgical treatment and transvenous embolization provide comparable efficacy and safety profiles [17]. Future studies may use standardized clinical outcomes to compare the long-term follow-up data from different surgical interventions.

Differences in demographic and anatomical characteristics between Type 1 and Type 3 patients observed in our cohort are generally consistent with previous studies. Schievink et al. reported a mean age of 43.4 years for Type 1 and 50 years for Type 3 patients [1], while Mamlouk et al. reported mean ages of 44.5 and 58.8 years, respectively [18]. Similarly, we observed that Type 1 leaks were more frequently located in the upper thoracic spine, whereas Type 3 leaks were more commonly found in the lower thoracic spine. However, given the small number of patients, these observations should be interpreted cautiously and considered exploratory.

Notably, only one of the 21 patients with available CSF opening pressure measurements had a pressure below 60 mmH2O. Although low CSF pressure has traditionally been regarded as a hallmark of intracranial hypotension, normal opening pressure is well recognized in contemporary SIH cohorts and is incorporated into current diagnostic frameworks [19].

The incidence of postoperative rebound headache in our cohort was 52.2%, which is higher than the 27–36% range reported in existing literature [20,21,22]. This discrepancy may be attributable to detection bias, as our patients underwent daily evaluation by a headache specialist, potentially allowing for the identification of milder cases. Alternatively, the delay from symptom onset to surgery in referred cases may have led to a chronic compensatory increase in CSF production [23,24], resulting in a more pronounced rebound phenomenon upon dural closure. Given that the diagnosis of rebound headache still relies heavily on clinical judgment [22], clearer criteria are needed to standardize reporting and management in future studies.

Our study has several limitations. First, its single-center, retrospective design limits the generalizability of our findings. Second, the study population was restricted to patients who underwent surgical treatment, representing a highly selected cohort of individuals with refractory SIH who had failed prior conservative or interventional treatments. In addition, the two subtypes entered the surgical cohort through different clinical pathways. Therefore, the study was not designed to provide causal or direct comparative inference between Type 1 and Type 3 leaks. These factors limit the generalizability of our findings to the broader SIH population. Third, our analysis focused exclusively on Type 1 and Type 3 leaks, and patients with Type 2 leaks were not included. Fourth, the number of patients with Type 3 leaks was small (n = 4). Although statistical differences between the groups were observed in several analyses, the small sample size limits the power for more detailed subgroup analyses. In addition, no formal correction for multiple comparisons was applied. Therefore, the risk of type I error should be considered when interpreting these exploratory subgroup findings. Fifth, the follow-up duration was limited to 2 months, which may not capture delayed recurrences or late complications. In addition, the exclusion of one patient who was unable to complete follow-up because of persistent frontotemporal brain sagging syndrome may have slightly overestimated favorable outcome rates. Sixth, the use of different radiological endpoints between subtypes may limit the comparability of outcomes. Finally, as surgical techniques inherently differ between subtypes, our study was not intended to compare procedural efficacy, and comparisons between groups should be interpreted cautiously. Despite these limitations, including the small number of patients with Type 3 SIH, our study provides real-world data regarding surgical management and short-term outcomes of carefully selected patients with SIH.

## 5. Conclusions

This study demonstrates that surgical treatment offers substantial clinical and radiological benefits for patients with SIH who do not improve with conservative management, particularly those with Type 1 ventral dural defects or Type 3 cerebrospinal fluid–venous fistulas. The application of recent advances in spinal imaging has enabled precise localization of CSF leaks, thereby improving surgical accuracy, increasing success rates, and enhancing patient safety. Further prospective, multicenter studies with larger cohorts are warranted to validate these results and refine surgical indications within this evolving diagnostic and therapeutic framework.

## Figures and Tables

**Table 1:** Demographic characteristics, clinical findings, and operative data.

	Patients with SIH (n = 23)	Type 1 Leak (n = 19)	Type 3 Leak (n = 4)	*p*-Value
Female sex, n (%)	14 (60.9%)	13 (68.4%)	1 (25%)	0.26
Age, years	42.9 (14.1)	38.2 (10.3)	65.3 (2.4)	<0.01
BMI, kg/m^2^	23.7 (3.3)	23.9 (3.3)	22.7 (3.6)	0.46
Symptom duration, days	135 (77–378)	239 (69–449)	110 (101–120)	0.46
Maximum headache intensity (NRS)	4.6 (2.8)	4.6 (2.8)	4.8 (2.7)	0.99
Daily upright time, hours	3 (1.5–8)	3 (2–8)	1 (0–5)	0.22
Preoperative EBP trials, n	3.9 (2.5)	4.2 (2.5)	2.5 (2.9)	0.46
Bern score	3.3 (2.7)	2.6 (2.3)	6.3 (2.2)	0.02
Organized SLEC, n (%)	–	14 (73.7%)	–	
Diagnostic modality identifying the leak				
DSM (%)	–	14 (73.7%)	2 (50%)	
Dynamic CTM (%)	–	16 (84.2%)	–	
Lateral decubitus CTM (%)	–	–	4 (100%)	
CSF opening pressure (mmH_2_O)	128.7 (52.3)	132.6 (56.6)	110.0 (18.3)	0.37
Leak location, n (%)				0.02
Upper thoracic	16 (69.6%)	15 (78.9%)	1 (25%)	
Mid thoracic	2 (8.7%)	2 (10.5%)	0 (0%)	
Lower thoracic	5 (21.7%)	2 (10.5%)	3 (75%)	
Intraoperative findings				
Operative time, min	141.9 (33.0)	151.7 (26.1)	95.5 (20.7)	<0.01
Estimated blood loss, mL	210.9 (202.9)	240.5 (211.6)	70 (35.6)	0.08
Dural defect size, mm	–	3 (2–3)	–	
Postoperative complications, n (%)				
Rebound headache	12 (52.2%)	12 (63.2%)	0 (0%)	0.04
Transient lower extremity weakness	3 (13.0%)	3 (15.8%)	0 (0%)	0.99
Hematoma requiring evacuation	1 (4.3%)	1 (5.3%)	0 (0%)	0.99

Values are presented as mean (standard deviation), median (interquartile range), or number (%). *p*-values indicate comparisons between the Type 1 and Type 3 groups. Leak levels were categorized as upper thoracic (C7/T1–T3/4), mid-thoracic (T4/5–T7/8), and lower thoracic (T8/9–T12/L1). SIH, spontaneous intracranial hypotension; BMI, body mass index; NRS, numeric rating scale; EBP, epidural blood patch; SLEC, spinal longitudinal epidural collection; DSM, digital subtraction myelography; CTM, computed tomography myelography; CSF, cerebrospinal fluid.

**Table 2:** Comparison of pre- and postoperative clinical and radiologic outcomes.

	Patients with SIH (n = 20)	Type 1 Leak (n = 16)	Type 3 Leak (n = 4)
Maximum headache intensity (NRS)			
Pre-surgical	4.6 (2.7)	4.6 (2.7)	4.8 (2.7)
Post-surgical	1.4 (1.6)	1.6 (1.6)	0.5 (1.0)
Daily upright time, hours			
Pre-surgical	3 (1–4.5)	3 (1.5–4.5)	1 (0–5)
Post-surgical	12 (5–16)	8.5 (5–16)	15 (13–16)
Bern score			
Pre-surgical	3.4 (2.8)	2.7 (2.5)	6.3 (2.2)
Post-surgical	0.9 (1.1)	0.9 (1.2)	0.8 (1.0)

Analysis was performed on 20 patients who completed the 2-month postoperative follow-up. SIH, spontaneous intracranial hypotension; NRS, numeric rating scale.

## Data Availability

The data presented in this study are available on request from the corresponding author. The data are not publicly available due to privacy and ethical restrictions.

## Supplementary Materials

The following supporting information can be downloaded at: https://www.mdpi.com/article/10.3390/jcm15134972/s1, Supplementary Video Clip S1. Intraoperative video showing the surgical repair of a Type 1 ventral dural tear at the T2–3 level. The defect is sealed using an autologous muscle graft reinforced with Hemopatch^®^. Supplementary Video Clip S2. Surgical ligation of a Type 3 CSF–venous fistula at the T12–L1 level. Intraoperative indocyanine green videoangiography confirms the identification and complete occlusion of the fistula.

## Abbreviations

The following abbreviations are used in this manuscript:

SIH	Spontaneous Intracranial Hypotension
CSF	Cerebrospinal Fluid
EBP	Epidural Blood Patch
NRS	Numeric Rating Scale
DSM	Digital Subtraction Myelography
SLEC	Spinal Longitudinal Extradural Collection
